# Autistic Adults Show Similar Performance and Sensitivity to Social Cues on a Visual Perspective Taking Task as Non-autistic Adults

**DOI:** 10.1007/s10803-022-05480-8

**Published:** 2022-02-19

**Authors:** Richard J. O’Connor, Joshua L. Plant, Kevin J. Riggs

**Affiliations:** Department of Psychology, University of Hull, Cottingham Road, Hull, HU16 4RS UK

**Keywords:** Autism, Visual perspective taking, Social cues

## Abstract

Autistic and non-autistic adults completed a visual perspective taking (VPT) task, reporting an object’s location from an actor’s perspective, or their own. On half the trials the actor looked at and reached for the object, and on half did not. Accuracy and reaction time were measured. In Experiment 1, both groups (N = 34, mean age = 24 years) responded slower when reporting the actor’s perspective, with no group differences in this effect. Experiment 2 included “other” VPT trials only. Both groups (N = 30, mean age = 25 years) showed sensitivity to the actor’s behaviour, more accurately reporting his perspective when he acted upon the object. No group differences were observed. In contrast to developmental studies, these experiments suggest similar VPT abilities in autistic and non-autistic adults.

**Supplementary Information:**

The online version contains supplementary material available at 10.1007/s10803-022-05480-8.

## Introduction

Our ability to work out what people can see, or how they see it, provides us with useful information during our social interactions with them. It helps us to work out what they might desire or wish to avoid, what knowledge or beliefs they might have, and what actions they are about to perform. In other words, visual perspective taking (VPT) is a key component in theory of mind (ToM)—our ability to reason about other people’s mental states in order to understand and predict their behavior (Mitchell & Riggs, 2000). It is likely that ToM and VPT share common cognitive processes as they both involve the simultaneous representation of two differing viewpoints. Thus, investigating VPT is likely to help us develop a deeper understanding of ToM processing in both children and adults.

VPT involves understanding that others can have a different perspective to one’s own: while for me my coffee cup is currently to my right, to someone stood opposite my desk and facing me that cup would appear to them to be on their left. Although this may seem a trivial example, the ability to understand perspectives different to one’s own sits at the core of successful social interactions.

Given that autistic individuals have difficulty understanding the beliefs and desires of other people this has led to a body of research investigating VPT in autistic children, adolescents and adults in order to gain a better understanding of their difficulties with social interaction and communication (for a review see Pearson et al., 2013). While some have suggested that autistic individuals display differences and impairments in VPT (e.g., Conson et al., 2015; Hamilton et al., 2009; Schwarzkopf et al., 2014), the overall evidence, particularly across development, is inconsistent (e.g., David et al., 2010; Santiesteban et al., 2015; Zwickel et al., 2011). The present two experiments explore VPT abilities in autistic adults, investigating whether differences in VPT previously observed in autistic adolescents (Conson et al., 2015) are also present in adulthood.

### Key Distinctions and Research Topics in VPT

Researchers have drawn a distinction between level 1 and level 2 perspective taking. Level 1 perspective taking refers to understanding what another agent can see. For example, understanding that I can see the text of the book I am reading, but you, sat opposite me, cannot, or understanding that while I might be able to see two pens on my desk an observer with an obstructed view might only be able to see one. Level 2 perspective taking refers to how the same object may appear differently to another agent. For example, understanding that the number on the table in front of us looks like a 6 to you, but a 9 to me, or that a cup that is on my right-hand side is located on the left-hand side of someone sat opposite me. Research into level 2 VPT has a long history going back to Piaget and Inhelder’s (1956) three mountains task where children of various ages were asked to judge how an array of three mountains would appear to another person. Level 1 and 2 VPT have different developmental time-courses with level 1 developing around 18–24 months and level 2 around 4–5 years (Flavell, 1977). Further, some have suggested that they rely on different cognitive processes, with level 2 VPT involving a mental transformation of one’s position in space whereas level 1 VPT may not require such a transformation (Surtees et al., 2013a, b). We return to the question of what cognitive strategies may underlie VPT level 2 judgements below.

A second distinction is between explicit, or deliberate, perspective taking and automatic, or spontaneous, perspective taking. In tasks measuring explicit perspective taking participants are directly instructed to report the perspective of another agent or respond to instructions given from another agent’s perspective, where that agent’s perspective is different from that of the participant. On such tasks errors are typically “egocentric intrusions”: participants mistakenly respond from their own perspective (e.g., Epley et al., 2004; Mazzarella et al., 2012). Further, adult participants are typically slower and more error prone when reporting the different perspective of another agent, than when participants either (a) report their own perspective, or (b) report the perspective of another when that perspective is the same as the participants’ own perspective (e.g., Samson et al., 2010; Surtees & Apperly, 2012).

In contrast to explicit perspective taking, a number of researchers have claimed that under certain circumstances people automatically, or spontaneously, process another agent’s perspective in the absence of any instructions or requirement to do so (e.g., Tversky & Hard, 2009; Samson et al., 2010). Tasks purportedly tapping *automatic perspective taking* typically involve conditions in which participants are instructed to respond from their own perspective (e.g., that they can see 3 dots) when there is another agent in the visual scene. If that participant has a perspective different to the agent (who might only be able to see 2 dots) participants tend to make more errors and are slower to respond when reporting their own perspective, relative to conditions in which the other agent’s perspective is consistent with that of the participant (e.g., Samson et al., 2010; Surtees & Apperly, 2012). This behaviour has been interpreted as evidence for an automatic perspective taking system: outside of their deliberate, conscious control participants are processing the perspective of the other agent, to the detriment of their ongoing task performance.

Other tasks provide evidence for *spontaneous perspective taking* of another agent’s perspective (e.g., Conson et al., 2017; Furlanetto et al., 2013; Mazzarella et al., 2012; Tversky & Hard, 2009; Zhao et al., 2015). For example, when presented with a picture of an object on a desk and simply instructed to report the location of that object when there is another person in the picture, a minority of participants spontaneously report the location from that person’s perspective, and not their own (Tversky & Hard, 2009). This suggests, at the very least, that other people’s perspectives are salient and spontaneously accessible, even if the exact process by which they are accessed (e.g., whether the result of an automatic mentalising process or not) is the subject of debate (Santiesteban et al., 2017; Heyes, 2014; Millett et al., 2020).

Researchers have also investigated whether the behaviour of an agent makes participants more likely to adopt that agent’s perspective. It has been suggested that seeing an agent interact with an object triggers the processing of the agent’s perspective in order to best understand and interpret their behaviour (e.g., Furlanetto et al., 2013; Tversky & Hard, 2009). For example, when an agent is looking at and reaching for an object and participants are required to report the location of that object, participants are more likely to report from the other agent’s perspective relative to control conditions in which the agent is not interacting with the object (Conson et al., 2017; Furlanetto et al., 2013; Mazzarella et al., 2012; Tversky & Hard, 2009). Further, when participants are required to explicitly report the perspective of the other agent, participants make fewer egocentric errors when the agent is reaching for the object compared to when the agent is not (Mazzarella et al., 2012). Overall these studies suggest that the behaviour of the agent influences the readiness with which their perspective is processed by participants, both in explicit and spontaneous perspective taking tasks.

### VPT Abilities in Autistic Adults: Automatic/Spontaneous VPT

Two studies have found evidence of similar automatic *level 1* VPT performance in autistic and non-autistic adults (Schwarzkopf et al., 2014; Tei et al., 2019), both using the dot-task of Samson et al. (2010). In these studies both participant groups showed slower and less accurate performance when reporting their own perspective when another agent in the visual scene had a different perspective (e.g., the participant could see three dots, but the agent only two), relative to trials on which the other agent’s perspective was consistent with the participant’s. A third study with the same task found similar effects for accuracy data, albeit no such effects for autistic participants on RT data (Doi et al., 2020). Only one study has measured automatic VPT *level 2* performance in autistic adults. Zwickel et al. (2011) found that both autistic and non-autistic adults were slower and less accurate when judging the position of an object (on the right, or on the left) from their own perspective when another agent had a different perspective, relative to trials when the agent’s perspective was the same as the participants. Overall, these studies suggest that automatic VPT level 1 and level 2 abilities are similar in both autistic and non-autistic adults.

### VPT Abilities in Autistic Adults: Explicit VPT

Studies of explicit VPT *level 1* abilities in adults have suggested differences between autistic and non-autistic participants, albeit with inconsistent patterns of findings. Schwarzkopf et al. (2014) found that, relative to control participants, autistic adults were slower to report another agent’s perspective compared to their own perspective. Conversely, when using the exact same task, Tei et al. (2019) found that while controls were slower to report the other agent’s perspective compared to their own, the autistic group did not show any such difference. This lack of consistency is also seen in studies measuring explicit VPT level 1 abilities using a different task: the director task (Eack et al., 2017; Santiesteban et al., 2015). Participants had to respond to instructions given by another person (the director). On some trials, successful responding required taking into account what the director could see (e.g., responding to “hand me the apple” when the participant could see three apples, but the director could only see one). Thus participants had to successfully adopt the perspective of the director in order to accurately respond on these trials. While Santiesteban et al. (2015) found no differences in performance between autistic and non-autistic individuals, Eack et al. (2017) found that autistic participants were less accurate than controls on trials which required perspective taking relative to trials that did not.

With regard to explicit VPT *level 2*, there appears to be only one study assessing autistic adults’ abilities (David et al., 2010). Participants had to judge whether an object appeared on the left or the right from another agent’s perspective. Both autistic and non-autistic adults were slower to report the other agent’s perspective relative to reporting their own, with no differences observed between the two groups (David et al., 2010). In contrast, individual difference studies, albeit with the general adult population and not clinical sub-groups, have suggested a negative relationship between VPT level 2 performance and Autism Quotient scores (Brunyé et al., 2012; Kessler & Wang, 2012). Overall, the extent to which VPT abilities in autistic adults differ from non-autistic controls is currently unclear (Pearson et al., 2013).

### Is VPT Influenced by the Actions of the Agent in Autistic Individuals?

No studies have investigated whether autistic adults display differences to non-autistic controls in the extent to which the behaviour of an agent triggers the processing of that agent’s perspective. We address this question in the research reported here. There is, however, one study investigating such differences in autistic adolescents (Conson et al, 2015). Participants had to judge the position (left or right) of a bottle, either from their own perspective or the perspective of an agent who was looking and/or reaching for the bottle. In general, autistic participants were slower than the non-autistic controls on all trial types and made more errors when responding from their own perspective. Furthermore, and most relevant to the present research question, for autistic participants the actions and gaze of another agent action did *not* appear to affect either accuracy or speed when judging the position of the object from either their own perspective, or the perspective of the other agent. For the non-autistic control group, however, the reaching action of the agent *did* affect performance. When the agent reached, non-autistic controls were more accurate and faster to judge the position of the bottle from the agent’s perspective. Conversely, when non-autistic controls judged from their own perspective, and the agent was reaching, then they were slower and made more errors.

Conson et al. (2015) interpreted their findings in terms of the different strategies that can be used to solve VPT level 2 tasks (Zacks & Michelon, 2005), suggesting that these strategies varied between the autistic and non-autistic participants (for similar recent suggestions regarding younger autistic children’s VPT abilities see: Pearson et al., 2016; Ni et al., 2021). One strategy, typically called an embodied egocentric transformation (EET: Pearson et al., 2016), involves imagining rotating one’s own body into the position of the other agent in order to judge their perspective. Another strategy would be to mentally rotate (MR) the relevant object or collection of objects itself through the required degree of angle in order to judge how it appears from a different perspective. Note the former strategy involves mentally transforming one’s own bodily position while keeping the relevant objects stationary, whereas the latter involves keeping the representation of one’s bodily position constant while rotating the relevant objects. These strategies might yield broadly similar levels of performance, yet they rely on different cognitive processes.

It has been suggested that seeing another agent reach and look at an object activates the processes underlying an EET strategy, and this explains why such stimuli enhance that processing of that other agent’s perspective in non-autistic populations (Conson et al., 2015; Furlanetto et al., 2013; Tversky & Hard, 2009). Observing another agent’s actions, or their possible intention to act as cued by their gaze towards a potential goal-object, is suggested to trigger the embodiment of their perspective in order to best understand and interpret their current and future actions (Furlanetto et al., 2013; Tversky & Hard, 2009). Conson et al. (2015) therefore proposed that if autistic individuals are *not* using an EET strategy, then their performance on a VPT level 2 task (judging the position—left or right—of a bottle) would *not* be sensitive to the presence of an agent who is looking and/or reaching towards the relevant object. Given that this is indeed what that authors found they suggested that autistic adolescents were adopting a different strategy, such as an MR strategy, while the non-autistic participants (whose VPT performance did display sensitivity to the agent’s behaviour) were adopting an EET strategy.

### VPT Performance Across Adolescence and Adulthood: Two Explanations and Predictions

While Conson et al. (2015) report a lack of sensitivity in adolescents to the agent’s behaviour in a VPT task, it is unclear if this reflects a persisting difference across the lifespan, or rather a developmental delay that will be overcome at some future point in development. Given that VPT level 2 abilities, both explicit and spontaneous, appear to be similar across autistic and non-autistic adults, how do we square these findings with those observed by Conson et al. (2015) with adolescents? One possibility is that the similar levels of performance seen in the two groups in adulthood (David et al., 2010; Zwickel et al., 2011) reflects the fact that in autism there is a developmental change in VPT level 2 abilities—for example, a transition from an MR strategy to the more “typical” EET strategy. In this case, one would expect that if autistic adults were tested on a VPT task similar to that used by Conson et al. (2015), then they *would* show sensitivity to the actions of another agent in their VPT performance, and no differences in performance when compared to non-autistic controls.

A second possibility is that although similar levels of performance have been seen between adult autistic and non-autistic groups on VPT level 2 tasks, this actually masks underlying differences in strategy preference and VPT abilities (cf. Pearson et al., 2016). Perhaps autistic adults, like autistic adolescents, do not use an EET strategy by default. Indeed, it is notable that the instructions used by David et al. (2010) specifically highlighted the use of an EET strategy to complete their explicit VPT level 2 task (“Imagine yourself standing in the position of the virtual character. It is very important to imagine your change in position!”). The explicit instructions to use an EET strategy when adopting the agent’s perspective could have altered strategy use away from a MR default, resulting in better than expected VPT performance in the autistic group, and hence no apparent differences between autistic and non-autistic adults (cf. Ni et al., 2021). Thus, we might expect—in a task that does *not* highlight the use of an EET strategy—that differences in explicit VPT level 2 performance might actually be observed between the groups of participants. Further, if autistic adults do *not* use an EET strategy by default, then they would *not* be predicted to show sensitivity to the actions of another agent in their VPT performance, as reported by Conson et al. (2015) for adolescents.

### The Present Study

In the following two experiments, we address the above two possible accounts by measuring autistic adults’ performance (accuracy and RT) on a VPT level 2 task using neutral instructions that did not highlight the use of any particular strategy. Further, we varied whether the other agent in the task looked at and reached for the object or not, in order to assess whether autistic adults show sensitivity in their performance on this VPT task to the agent’s behaviour. It should be noted that across both experiments we compared two “social cue” conditions only: a baseline no-cue condition in which the agent neither looked nor reached; and a cue condition in which the agent both looked and reached. Given that we were not interested in the relative salience of looking versus reaching for triggering adoption of another perspective, but rather the sensitivity of autistic individuals’ VPT performance to the presence of such cues in general, we did not adopt “look but not reach” and “reach but not look” conditions as used in previous work (e.g., Conson et al., 2015; Mazzarella et al., 2012). By comparing a minimal (actor present, but with no cues) to a maximal (both cues) condition only, we reduced the number of required conditions for the key comparison of interest, and thus reduced participant fatigue and increased statistical power.

## Experiment 1

Experiment 1 presented participants with both “self” and “other” perspective judgement trials in which they had to judge the position of a bottle (on the left or on the right) on a table. Another agent—a male actor—sat on the opposite side of the table, such that from his perspective the bottle was always on a different side to that of the participant. The actor either looked at and reached for the bottle (“cue” condition) or did not (“no-cue” condition). Experiment 1 tested two hypotheses. First, if autistic adults do have impaired VPT level 2 performance, and the results of David et al. (2010) are an artefact due the particular instructions used, then one would predict that the autistic group would perform worse specifically on the “other” judgements relative to non-autistic controls. Second, if autistic adults do not adopt an EET strategy by default, then one would predict no difference in their performance between the cue and no-cue conditions on either “self” or “other” judgements, as has been observed in autistic adolescents (Conson et al., 2015). For non-autistic controls, however, the cue condition was predicted to enhance performance when making “other” judgements, and reduce performance when making “self” judgements.

## Methods

### Participants

Twenty participants with a diagnosis of an autism spectrum condition from a psychiatrist or clinical psychologist, based on the International Classification of Diseases (ICD-10) criteria, completed the study. Autistic participants were undergraduates at the University of Hull and studied a range of academic subjects, drawn from the faculties of health, science, arts and humanities. They were invited to take part in the study via the Disability Services at the University and the National Autistic Society. From these 20 autistic participants, three participants were excluded from the final data analysis. One participant was excluded because of abnormally slow reaction times (their average reaction time exceeded 4000 ms: almost 2000 ms slower than then next slowest participant). Two further participants were excluded because of a high number of incorrect responses, with an error rate of 50% or more in at least one condition.

The final autistic group therefore comprised 17 participants (8 females and 9 males; mean age = 23 years). This group was compared to an age-and gender-matched control group of 17 non-autistic participants (8 females and 9 males; mean age = 24). There was no significant difference in age between the autistic group and the control group, (t(32) = .24, p = 0.82). Participants in the control group were psychology undergraduates and were invited to take part in the study through the University of Hull, Department of Psychology Research Participation Scheme. All participants completed the 50-item Autism Quotient (AQ) questionnaire (Baron-Cohen et al., 2001). As expected, the autistic group’s AQ scores (mean = 35) were significantly higher than the control group (mean = 15), t(32) = 6.21, p < .001. All participants had normal or corrected-to-normal vision and all participants were right-handed.

### Apparatus and Stimuli

**Fig. 1:**
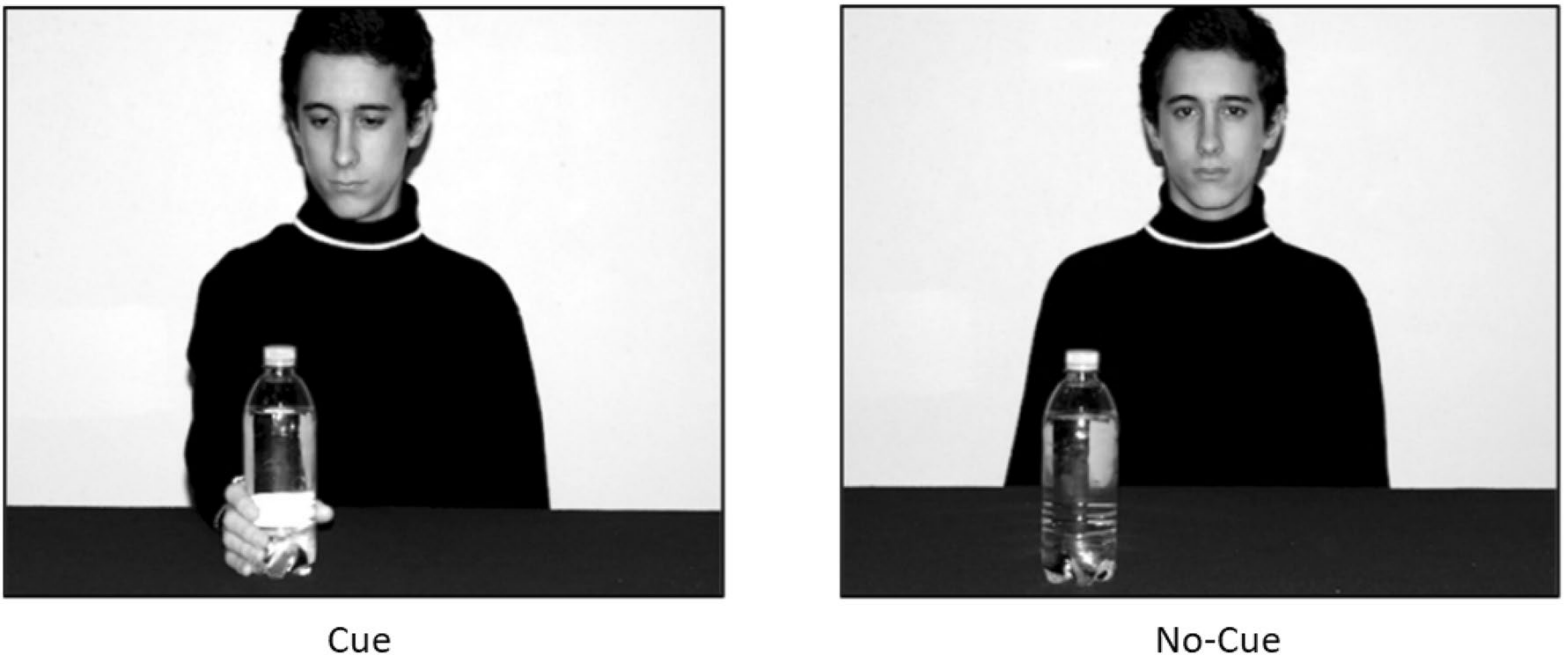
Example stimuli used in Experiment 1, as used in Conson et al. (2015)

The perspective-taking task was presented on a 19-inch PC-desktop, viewed at a distance of approximately 50 cm, using OpenSesame version 3.2.5 (Mathôt et al., 2012). The stimuli (see Fig. 1) used in the task were four grayscale photographs in which a male actor sat at a table, with a water bottle appearing either on the left or on the right. The actor either both looked at and reached for the bottle (cue condition), or looked straight at the camera and did not reach for the bottle (no-cue condition). These stimuli were the same as those used in Conson et al. (2015) Experiment 1, using their “yes-gaze/yes-grasp” and “no-gaze/no-grasp” conditions respectively. The stimuli measured 17 × 13.5 cm when displayed on the computer screen.

### Design and Procedure

The experiment had a 2 × 2 × 2 mixed design with perspective (self vs. other) and social cue (no-cue vs. cue) as repeated-measures factors and group (autistic vs. control) as a between-subjects factor. Participants were instructed to identify the location of the bottle in each photograph, responding either from their own perspective (self) or from the perspective of the actor in the photograph (other). The instructions made no reference to how participants should approach the task, simply stating: “Your task is to view pictures and identify the location of a bottle in each picture, QUICKLY and ACCURATELY. When the word 'self’ is presented on the screen, followed by the picture, you should respond from your own perspective. When the word 'other’ is presented on the screen, followed by the picture, you should respond from the actor’s perspective.”. Following Mazzarella et al. (2012) and Conson et al. (2015), participants used a QWERTY keyboard to make their responses, pressing “B” for left and “H” for right using their right-hand. This procedure for responding was chosen in order to reduce spatial compatibility effects.

In each trial in the main experimental block, participants were first presented with a fixation cross, centrally presented for 800 ms, followed by either the word “self” or “other”, centrally presented for 1000 ms. After the presentation of this word, a second fixation cross was presented for 800 ms, followed by one of the four photographs. Photographs remained on-screen until the participant gave their response. Accuracy and RT were recorded for each trial.

Prior to the main experimental block of trials, participants first completed a set of 16 practice trials (8 self trials and 8 other trials, using the same pictures as in the experimental block). In the practice trials, the word (“self” or “other”) was presented for 2000 ms. After the 16 practice trials, participants received feedback on their performance (average accuracy and RT, presented on the computer screen). These 16 trials were removed from the statistical analysis.

Following the practice trials, participants completed 80 trials in the main experimental block. Self and other trials were presented equally within the block (40 of each), as were cue and no-cue trials (40 of each: 20 per self/other perspective). Whether the bottle appeared on the left or the right was also counter-balanced within each combination of perspective x social cue. Therefore each photograph stimulus appeared 20 times overall, 10 times for each perspective. The order of trials within the block was randomised, but with a selection rule such that the same perspective was not presented more than two consecutive times. Participants were given the opportunity to take a break halfway through the block.

## Results

**Fig. 2:**
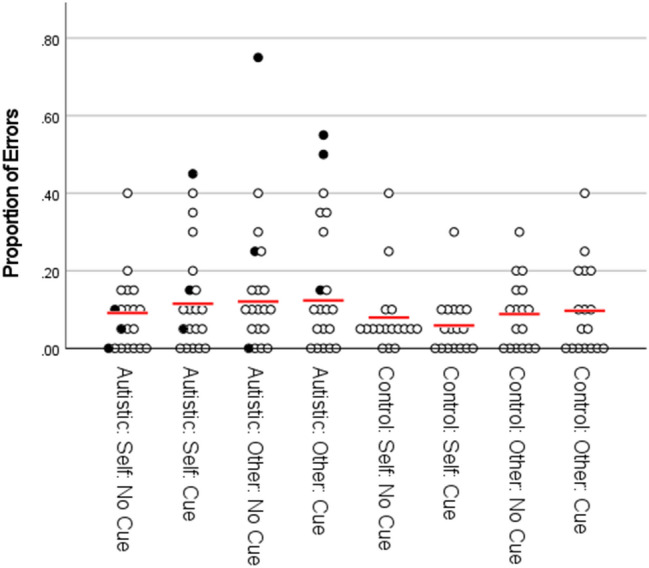
Proportion of errors by group and condition for Experiment 1. NB: figure includes data points for participants identified as outliers in the main text (filled circles), including one participant identified as an outlier on the basis of their RT data. Red lines represent means calculated with outliers excluded

**Fig. 3:**
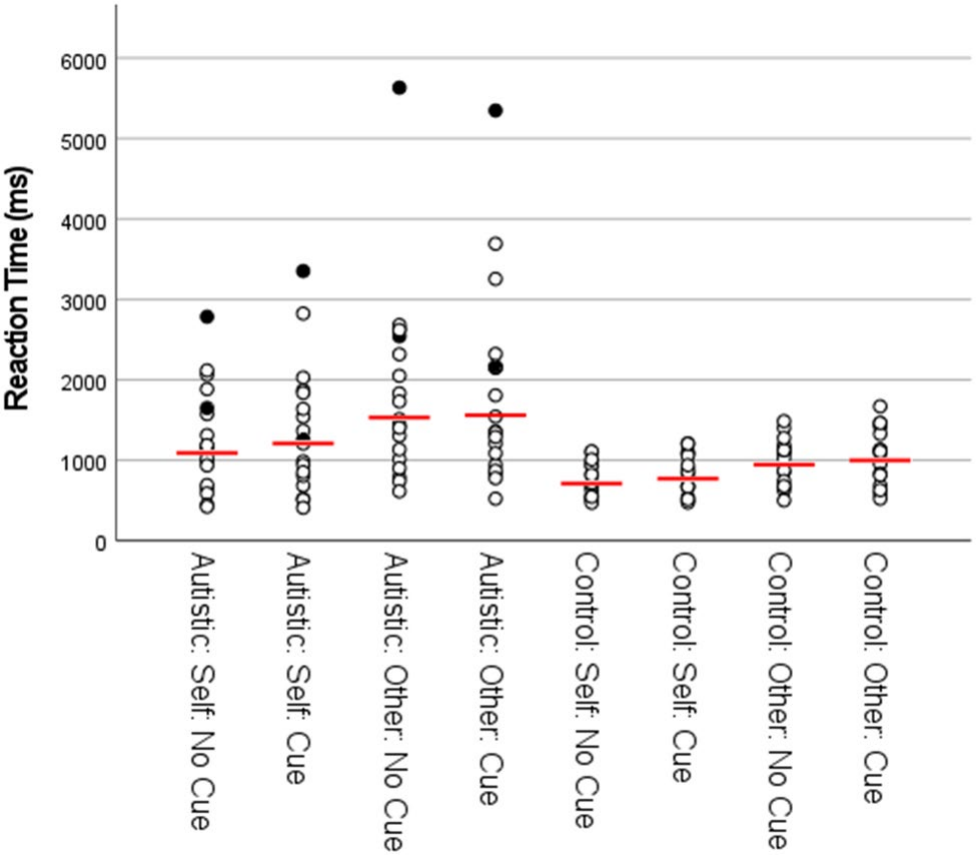
Reaction Time by group and condition for Experiment 1. NB: figure includes data points for participants identified as outliers in the main text (filled circles), including one participant identified as an outlier on the basis of their ER data. Data from one participant outlier is not shown due to too few correct trials recorded for one or more conditions. Red lines represent means calculated with outliers excluded

As can be seen in Figs. 2, 3, 5 and 6, in both Experiment 1 and Experiment 2, the error rate and reaction time data violated assumptions of normality or homogeneity of variance in some instances. Given that ANOVA methods have been demonstrated to be robust both with non-normally distributed data (Berkovits et al., 2000; Blanca et al., 2017; Schmider et al., 2010) and under conditions of heterogeneity when sample sizes are equal (Blanca et al., 2018; von Ende, 2001), we continue to report parametric statistics below. Where significant repeated-measures effects are observed we also report separate comparisons within each group, which as well as being of theoretical importance also controls for the risk of inflated F-values due to between-group heterogeneity (Hertzog & Rovine, 1985). Further, we also provide non-parametric analyses of the data from both experiments in the supplementary information. In all cases the findings reported in the main text ANOVAs are confirmed by non-parametric statistics.

For Experiment 1 a 2 × 2 × 2 ANOVA was conducted, with perspective (self vs. other) and social cue (no-cue vs. cue) as repeated-measures factors and group (autistic vs. control) as a between-subjects factor. Separate analyses were run for error rate (ER) and for reaction time (RT).

### Error Data

For the ER data (see Fig. 2), the 2 × 2 × 2 ANOVA did not show any significant differences in performance across any of the factors. There were no significant main effects for perspective, F (1, 32) = 1.64, p = .21, η_p_^2^ = .049, social-cue, F (1, 32) = .078, p = .78, η_p_^2^ = .002, or group, F (1, 32) = 1.16, p = .29, η_p_^2^ = .035. There were also no significant two-way interactions between group and perspective, F (1, 32) = .018, p = .90, η_p_^2^ = .001, group and social-cue, F (1, 32) = .53, p = .47, η_p_^2^ = .016, or perspective and social-cue, F (1, 32) = .055, p = .82, η_p_^2^ = .002, nor was there a significant three-way interaction between group, perspective and social-cue, F (1, 32) = 1.76, p = .19, η_p_^2^ = .052.

### RT Data

All trials that were incorrect (9.7% of the data) and trials that were above or below 3 SD of the participant overall mean RT (1.2% of the data) were removed from the RT analysis. For the remaining RT data (see Fig. 3), the 2 × 2 × 2 ANOVA showed a significant main effect of group, F (1, 32) = 8.60, p = .006, η_p_^2^ = .21, with autistic participants responding slower overall than control participants. There was also a significant main effect of perspective, F (1, 32) = 31.95, p < .001, η_p_^2^ = .50, with responses from the other perspective slower overall than responses from the self perspective. There was no significant main effect of social cue, F (1, 32) = 1.85, p = .18, η_p_^2^ = .055. Further, there were no significant two-way interactions between group and perspective, F (1, 32) = 2.17, p = .15, η_p_^2^ = .064, group and social-cue, F (1, 32) = .042, p = .84, η_p_^2^ = .001, or perspective and social-cue, F (1, 32) = .65, p = .43, η_p_^2^ = .020, nor was there a significant three-way interaction between group, perspective and social-cue, F (1, 32) = .48, p = .49, η_p_^2^ = .015.

While there was no significant interaction between group and perspective, paired t-tests were conducted separately within each group to check that the difference in RT between self and other perspectives was significant within both the autistic and control groups. When collapsing mean RTs across both levels of the social-cue factor, responses from the other perspective were slower than responses from the self perspective within both the autistic group (mean diff = 382 ms), t (16) = 3.66, p = .002, d = .89 and the control group (mean diff = 226 ms), t (16) = 6.27, p < .001, d = 1.52.

## Discussion

This experiment addressed two hypotheses. First, whether autistic adults would show worse performance, relative to non-autistic controls, specifically on the “other” judgements on a VPT level 2 task, with neutral instructions that did not emphasise the use of any particular strategy. Here we observed no such differences between the groups: both groups showed slower performance when judging another person’s perspective relative to when judging their own perspective. There was no evidence that the size of this effect varied between the two groups. This is consistent with performance observed by David et al. (2010), ruling out the possibility that performance seen in David et al. (2010) between their autistic and non-autistic groups was caused by instructions emphasising an EET strategy.

While both groups in Experiment 1 were slower on “other” judgements compared to “self” judgements, the autistic group was slower overall when compared to the non-autistic group on both types of judgement. Longer response times in general are not typically observed in autistic adults on VPT tasks (e.g., David et al., 2010; Schwarzkopf et al., 2014; Tei et al., 2019), though in other domains it has been suggested that they display a slower, more deliberative response style (e.g., Brosnan et al., 2016). It is plausible that the general slowing in response times by the autistic group in Experiment 1 reflects differences in domain-general processes, such as executive functions. Our task required participants to switch between “self” and “other” judgements: in such a design participants are required to monitor on every trial which type of judgement they are required to make, with the two different judgements requiring opposite responses (when the bottle was on the left for a “self” judgement, it was therefore on the right for an “other” judgement). This would have drawn upon participants’ executive functions, such as cognitive flexibility, inhibition and working memory, which are known to be impaired in autistic individuals (for a recent meta-analysis, see Demetriou et al., 2018). We return to this issue in the general discussion.

Experiment 1 also aimed to test whether autistic adults show sensitivity to the actions of another agent during a VPT task. However, the data here are unable to address that hypothesis. While the autistic group did not show any differences in performance according to whether the actor looked and reached for the object or not, *the non-autistic control group did not display any predicted differences* across the social cue conditions either. This lack of an effect of social cue even in the control group is in contrast to the effects reported by Mazzarella et al. (2012) and Conson et al. (2015) using a similar type of task. This failure to replicate those effects requires further consideration.

One explanation could be that Experiment 1 used interleaved “self” and “other” trials within blocks. This practice is common in other studies of VPT, both in studies with non-autistic adults alone (e.g., Samson et al., 2010) and in those with autistic participants (e.g., Schwarzkopf et al., 2014; Tei et al., 2019; Doi et al., 2020). However, it is notable that those studies that specifically assess the effects of an agent’s looking and reaching have either separately blocked the “self” and “other” trials (Conson et al., 2015; Mazzarella et al., 2013), or used only one type of judgement within a single experiment (Mazzarella et al., 2012). As noted above, interleaving “self” and “other” trials in Experiment 1 would have placed demands upon participants’ cognitive flexibility, inhibition and working memory. Such demands may have led both groups of participants to neglect processing of the social cues, and therefore led to the lack of any effect of those cues upon their performance. Experiment 2 addressed this possible explanation.

## Experiment 2

In Experiment 2, participants completed the “other” judgement condition only, thus following the design of Mazzarella et al. (2012), who first reported the effects of another agent’s actions upon explicit perspective taking in adults. We did not include a separate “self” judgement condition in Experiment 2 for both practical and theoretical reasons. First, in order to follow the design of Mazzarella et al. (2012) the inclusion of an additional “self” judgement condition would have required a between-subjects design. Given the difficulties inherent in recruiting autistic participants, we would have faced substantial difficulties recruiting sufficient numbers for such a design. Second, where differences have been reported between autistic and non-autistic groups on VPT performance, this has tended to be in conditions requiring explicit perspective taking (i.e., “other” judgements: see Schwarzkopf et al., 2014). Autistic and non-autistic adults tend to show similar automatic VPT performance (i.e., effects of another agent’s conflicting perspective upon “self” perspective judgements: see Zwickel et al., 2011; Schwarzkopf et al., 2014; Tei et al., 2019). Experiment 2 therefore included only the “other” judgement condition, in order to test whether autistic adults show sensitivity to the actions of another agent when making such VPT judgements.

## Methods

### Participants

Sixteen participants with a diagnosis of an autism spectrum condition from a psychiatrist or clinical psychologist, based on the International Classification of Diseases (ICD-10) criteria, completed the study. None had participated in Experiment 1. They were undergraduates at the University of Hull and studied a range of academic subjects, drawn from the faculties of health, science, arts and humanities. They were invited to take part in the study via the Disability Services at the University, the National Autistic Society and a local autism charity. From these 16 autistic participants, one participant was excluded from the final data analysis because they had misunderstood the task.

The final autistic group therefore comprised 15 participants (10 females and 5 males; mean age = 25 years). This group was compared to an age-and gender-matched control group of 15 non-autistic participants (10 females and 5 males; mean age = 26). There was no significant difference in age between the groups, (t(28) = .06, p = .95). Participants in the control group were psychology undergraduates invited to take part in the study through the University of Hull, Department of Psychology Research Participation Scheme. None had participated in Experiment 1. All participants were screened with the 50-item Autism Quotient (AQ) questionnaire (Baron-Cohen et al., 2001). As expected, the autistic group’s AQ scores (mean = 36) were significantly higher than those of the control group (mean = 13), t(28) = 7.40, p < .001. All participants had normal or corrected-to-normal vision and all participants were right-handed.

### Apparatus and Stimuli

**Fig. 4:**
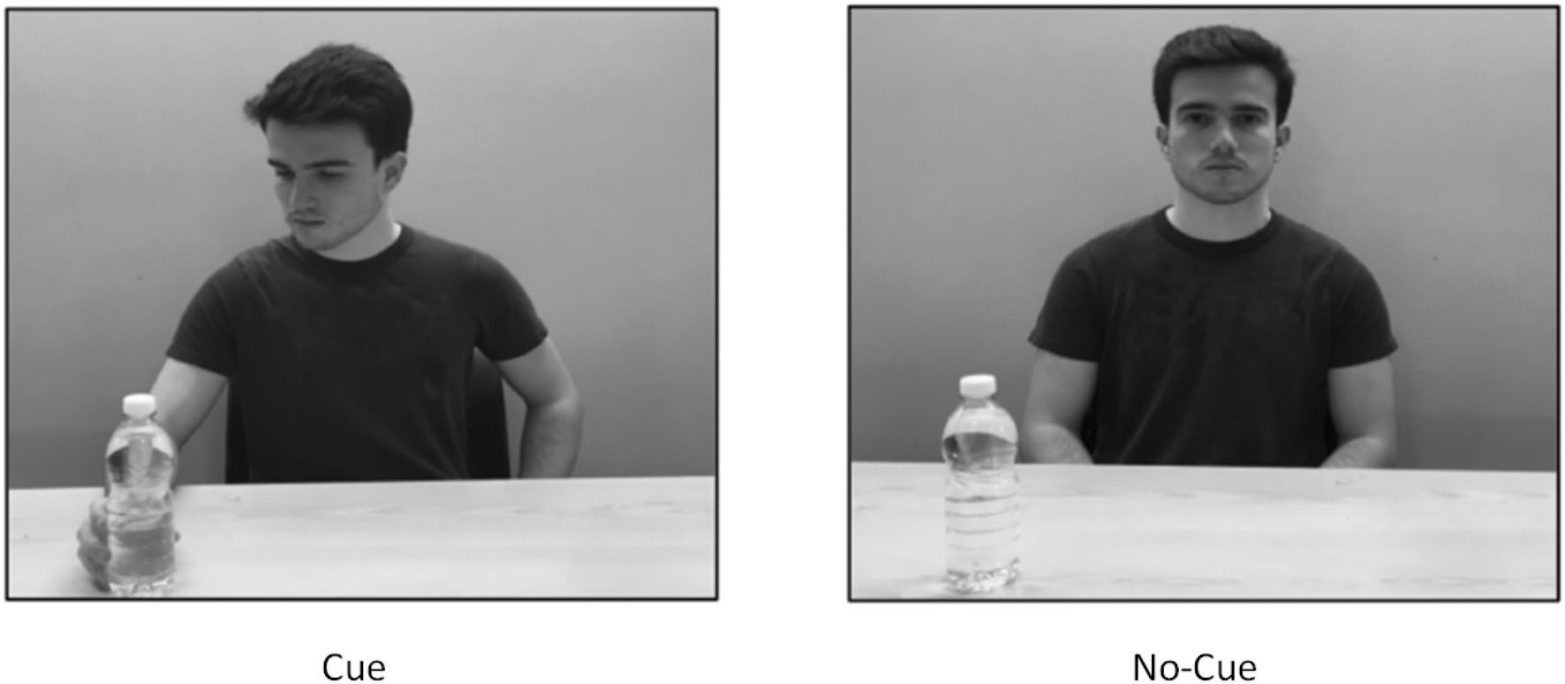
Example stimuli used in Experiment 2

The perspective-taking task was presented using the same computer, screen and software as in Experiment 1. As in Experiment 1, the stimuli used in the task (see Fig. 4) were four grayscale photographs in which a male actor sat at a table, with a water bottle appearing either on the left or on the right. The actor either both looked at and reached for the bottle (cue condition), or looked straight at the camera and did not reach for the bottle (no-cue condition). The stimuli measured 23 × 18 cm when displayed on the computer screen.

### Design and Procedure

The experiment had a 2 × 2 mixed design with social cue (no-cue vs. cue) as a repeated-measures factor and group (autistic vs. control) as a between-subjects factor. Participants were instructed to identify the location of the bottle in each photograph, responding from the perspective of the actor in the photograph (i.e., all trials were “other” perspective trials). Participants used a QWERTY keyboard to make their responses, pressing “B” for left and “H” for right using their right-hand.

In each trial, participants were first presented with a fixation cross, centrally presented for 800 ms, followed by one of the four photographs. Photographs remained on-screen until the participant gave their response. Participants were instructed to respond as quickly and as accurately as they could. Accuracy and RT were recorded for each trial.

Prior to the main experimental block of trials, participants first completed a set of 8 practice trials. After these 8 practice trials, participants received feedback on their performance (average accuracy and RT, presented on the computer screen). These 8 trials were removed from the statistical analysis.

Following the practice trials, participants completed 60 trials in the main experimental block. Cue and no-cue trials were presented equally within the block (30 of each). Whether the bottle appeared on the left or the right was counter-balanced within each social cue condition. Therefore each photograph stimulus appeared 15 times overall. The order of trials within the block was randomised. Participants were given the opportunity to take a break halfway through the block.

## Results

A 2 × 2 ANOVA was conducted, with social cue (no-cue vs. cue) as a repeated-measures factor and group (autistic vs. control) as a between-subjects factor. Separate analyses were run for error rate (ER) and for reaction time (RT).

### Error Data

**Fig. 5:**
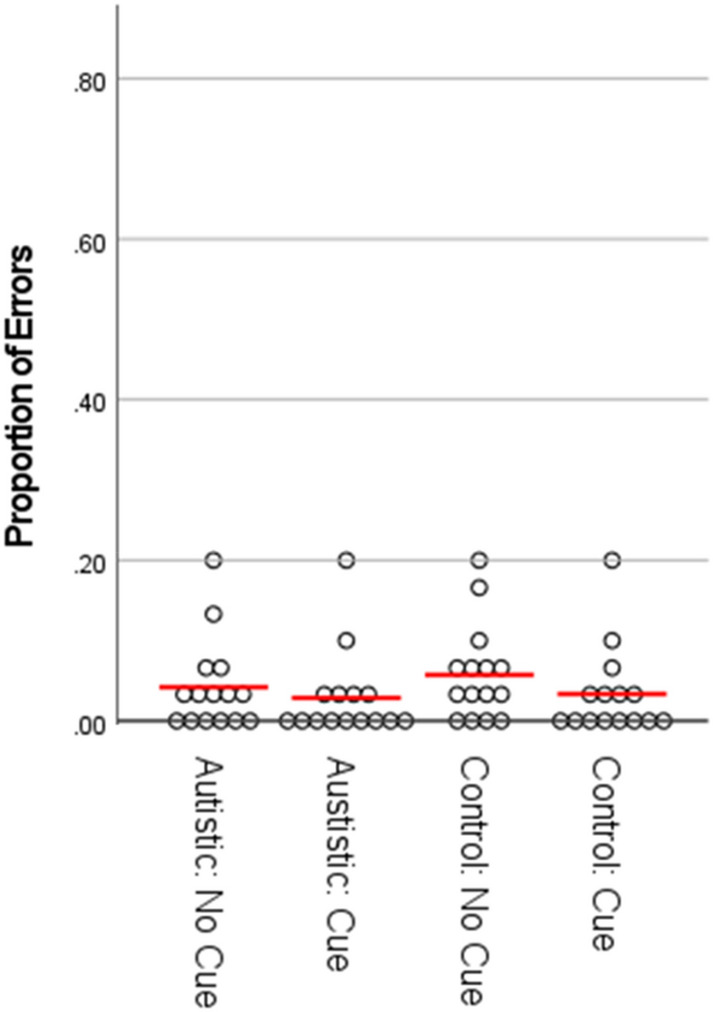
Proportion of Errors by group and condition for Experiment 2. Red lines represent means

For the ER data (see Fig. 5), the 2 × 2 ANOVA showed a significant main effect of social-cue, F (1, 28) = 7.81, p = .009, η_p_^2^ = .22, with fewer errors in the cue condition than in the no-cue condition. There was no significant main effect of group, F (1, 32) = .26, p = .62, η_p_^2^ = .009, and no significant interaction between group and social-cue, F (1, 32) = .68, p = .42, η_p_^2^ = .024.

While there was no significant interaction between group and social-cue, paired t-tests were conducted separately within each group to check whether the difference in ER between cue and no-cue conditions was significant within both the autistic and control groups. This difference was marginally significant within both the autistic group (mean diff = 0.013), t (14) = 2.10, p = .054, d = .54, and the control group (mean diff = 0.024), t (14) = 2.05, p = .060, d = .53.

### RT Data

**Fig. 6:**
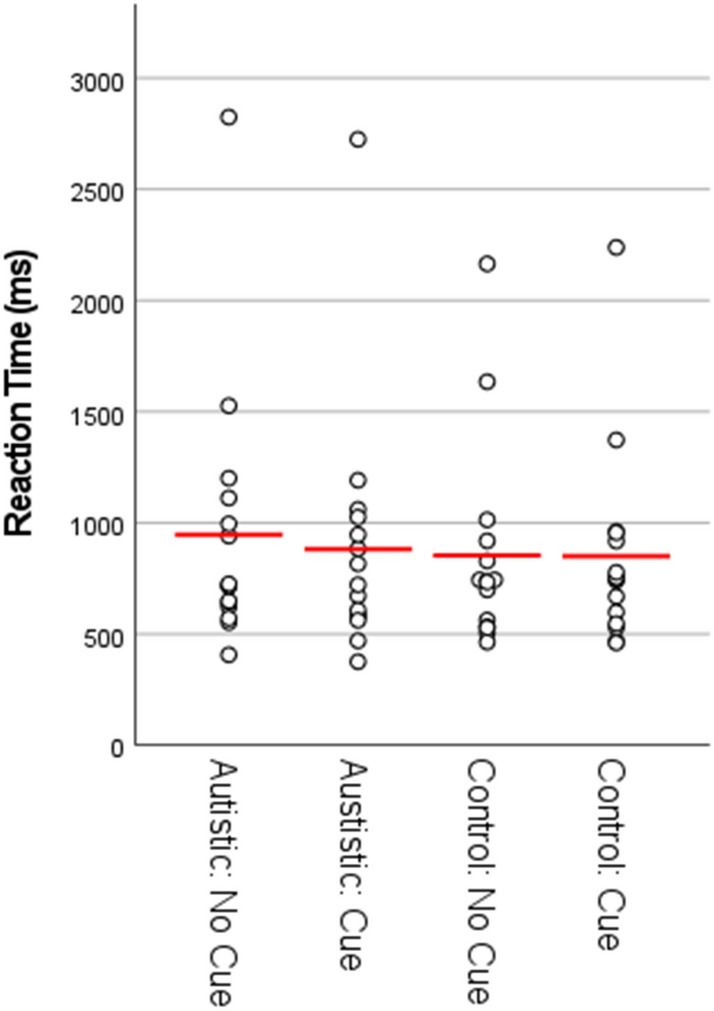
Reaction Time by group and condition for Experiment 2. Red lines represent means

All trials that were incorrect (4.1% of the data) and trials that were above or below 3 SD of the participant overall mean RT (2.2% of the data) were removed from the RT analysis. For the remaining RT data (see Fig. 6), the 2 × 2 ANOVA did not show any significant differences in performance across any of the factors. There were no significant main effects for social-cue F (1, 28) = 1.98, p = .17, η_p_^2^ = .066, or group, F (1, 28) = .11, p = .74, η_p_^2^ = .004, and there was no significant interaction between group and social-cue, F (1, 28) = 1.48, p = .23, η_p_^2^ = .050.

## Discussion

In Experiment 2 participants completed “other” perspective judgement trials only, following the design of Mazzarella et al. (2012). A main effect of social cue was observed across all participants: when the actor looked at and reached towards the object, participants made fewer egocentric errors when adopting his perspective. This matches the findings of Mazzarella et al. (2012), who also found an effect of social cue upon error rate, but not RT. There was no evidence for any differences in performance between autistic and non-autistic participants, either in terms of accuracy or RT. Together, these findings are consistent with our explanation of performance in Experiment 1 above: when the task demands of switching between perspectives were removed in Experiment 2, autistic participants no longer showed any differences in RT relative to the control group, and across both groups an effect of social cue emerged.

The effect of social cue on accuracy was marginally significant within both separate groups of participants. If sensitivity to another agent’s actions when adopting their perspective is to be interpreted as a hallmark of using an EET strategy, as has been claimed by Conson et al. (2015), then Experiment 2 suggests that autistic adults, like non-autistic adults, do adopt an EET strategy on this type of VPT task.

A potential challenge to the conclusion presented here should be addressed. As an anonymous reviewer pointed out, it might be argued that attentional cueing mechanisms were responsible for the social cueing effect observed in Experiment 2—especially since the social cue was arguably more salient than the cue used in Experiment 1 (the reaching of the actor was more obviously seen). Perhaps this attentional cueing, coupled with a participant strategy of ‘respond from my own perspective but press the opposite button’ might have given rise to the main of effect of social cueing. There are, however, two reasons to doubt such an interpretation. First, we did not find, nor did previous research using almost identical stimuli (Mazzarella et al, 2012), that grasping actions facilitated faster detection of a bottle—there was no effect of social cue in our reaction time data. Second, even if the actor grasping did facilitate attentional cueing in the absence of a reaction time advantage, the error rate data speak against this interpretation. Our participants made *fewer* errors on social-cue trials than on no-cue trials. If participants were minded to respond from their own perspective (but deliberately pressing the opposite button), then we would surely predict that an attentional cue to a specific location *from their own perspective* would lead to more errors on social-cue trials, or at the very least no difference in error rates across trial types. We would not predict fewer errors. We therefore suggest, in agreement with the wider literature using this type of laterality judgement VPT task (e.g., Conson et al., 2015, 2017; David et al., 2010; Mazzarella et al., 2012), that participants were adopting the perspective of the actor during this task and this process was facilitated by the social cue of the actor reaching for the object.

## General Discussion

Overall, Experiments 1 and 2 found no evidence that on VPT level 2 tasks autistic adults show different levels of performance, or adopt different strategies, compared to non-autistic controls. Both groups showed slower performance on “other” judgements compared to “self” judgements in Experiment 1, as has been previously found by David et al. (2010). While David et al. (2010) used explicit instructions to adopt an EET strategy, in Experiment 1 the instructions to participants were neutral. That the results of Experiment 1 and David et al. (2010) align suggests that the lack of any group differences between autistic and non-autistic adults observed in David et al. (2010) cannot be explained by autistic participants benefiting from the explicit guidance to use an EET strategy. Instead, the results of Experiment 1 and David et al. (2010) taken together suggest that explicit VPT level 2 abilities do not differ in autistic and non-autistic adults.

In Experiment 1 autistic participants responded slower than non-autistic controls in both “self” and “other” conditions. We suggested that this slower responding was due to the executive demands placed upon participants by the interleaving of “self” and “other” trials. In Experiment 2 such demands were removed by including “other” trials only. Here no group differences in RT were observed, consistent with our explanation for the general slower responding observed in Experiment 1.

Experiment 2 further addressed whether autistic adults adopt an EET strategy to solve this type of VPT task. Overall, participants in Experiment 2 showed better perspective taking when the actor looked at and reached for the object, with no evidence that this effect differed between both groups of participants. It has been claimed that seeing another agent direct an action towards an object triggers a spontaneous embodiment of their perspective in an attempt to interpret and respond to their actions (Furlanetto et al., 2013; Tversky & Hard, 2009). As such, better VPT performance when observing another agent act has been interpreted as evidence that participants are using an EET strategy on a VPT task (Conson et al., 2015). Indeed, if participants were solving the VPT task in Experiment 2 by instead mentally rotating the table with the bottle on it and then responding according to where the bottle now appeared from their own perspective, then it is difficult to explain why such a process would benefit from observing the actor reach for the bottle. If both autistic and non-autistic participants do adopt the same strategy on this type of VPT task, then this is consistent with the overall lack of group differences observed in both Experiment 1 and David et al. (2010).

Our findings from Experiment 2 differ to those reported by Conson et al. (2015) who found little evidence that the VPT performance in autistic adolescents was sensitive to the actions of the other agent. They interpreted their findings as evidence that autistic adolescents do not use an EET strategy on this type of task. We consider two possible explanations for the difference between their findings and ours.

First, this difference may reflect a genuine developmental change in VPT abilities. Plausibly, autistic children and adolescents may initially adopt a non-EET strategy such as MR, and later in development shift to an EET strategy. Alternatively, though not mutually exclusive to a shift in strategy-use, developmental change may occur in the ability to process the actions of other agents (Kaiser & Pelfrey, 2012), allowing such actions to be processed as relevant to perspective taking. Other closely related theory of mind abilities appear to show similar patterns, in which initial differences in an ability between autistic and non-autistic children reduce with age. While performance on classic theory of mind tasks is different in young autistic children compared to non-autistic controls (e.g., the false belief task: Baron-Cohen et al., 1985), these children and adolescents do show developmental changes in theory of mind abilities as they get older (Scheeren et al., 2013; Steele et al., 2003), with the pattern of these changes suggesting a delayed, rather than a deviated, course of development, at least in high-functioning autism (Hoogenhout & Malcolm-Smith, 2014).

Second, it might be the case that the findings of Conson et al. (2015) underestimated VPT performance in autistic adolescents. Conson et al. (2015) used five different cue conditions, and participants completed two different tasks (i.e., “self” and “other” judgements), albeit blocked, where in any given block they had to perform the opposite response to that in the previous block. In comparison to Experiment 2, therefore, the stimulus variability and task demands in Conson et al. (2015) would have been higher. While the non-autistic group did still demonstrate the expected effect of actor actions upon their VPT performance, it could have been the case that the autistic group were more affected by those task demands. Given the known impairments in executive functions in autistic individuals (Demetriou et al., 2018), resolving those task demands could have reduced available attentional resources for processing the actions of the actor. If one were to use a simplified design in which participants responded to fewer cue conditions and completed only one task with those stimuli, as in Experiment 2, it is possible that autistic adolescents might then display sensitivity to the actions of the actor in their VPT performance.

While Experiment 1 and 2 found no evidence for group differences on VPT level 2 performance, we would, however, caution against concluding that in all instances autistic adults display similar VPT level 2 abilities to non-autistic adults. While the task used in Experiment 2 was one adopted from previous VPT research (Mazzarella et al., 2012), it is a relatively simple task with low task demands, requiring participants to repeat the same judgement on a limited set of stimuli. As such, the simplicity and repetitive nature of this task might have allowed autistic participants to consciously adopt an efficient EET strategy that they otherwise might not have done with a more complicated or less repetitive task. For example, VPT tasks requiring a single, spontaneous judgement without instruction to take a particular perspective, such as that used by Tversky and Hard (2009), might reveal differences in performance between autistic and non-autistic adults.

Moreover, the experiments reported here, and in both David et al. (2010) and Zwickel et al. (2011) all use the same type of VPT level 2 decision: judging whether an object appears on the left or the right. It has been questioned whether this type of spatial judgement involves the same perspective taking processes as other VPT level 2 tasks (Pearson et al., 2013; but see Surtees et al., 2013a, b). Different tasks, such as those requiring judgement of an ambiguous stimulus (e.g., the number ‘6’) that can be perceived differently from different perspectives (e.g., Zhao et al., 2015), might produce different results to those reported here. Further, it remains to be seen whether there are robust, consistent differences in the VPT level 1 abilities of autistic adults (Eack et al., 2017; Santiesteban et al., 2015; Schwarzkopf et al., 2014; Tei et al., 2019). Finally, while we have focused here on the effects of looking and reaching upon VPT, other researchers have investigated the role that an agent’s emotions might play in triggering perspective taking (Zwickel & Müller, 2010), including in other participant groups with known theory of mind deficits (e.g., alcoholics: Cox et al., 2016, 2018). We would encourage future research that explores the performance of autistic individuals across the full range of VPT tasks used in the literature, before drawing firm conclusions as to whether their VPT abilities do, or do not, differ from non-autistic controls.

It is also important to consider the extent to which performance of our autistic groups in Experiments 1 and 2 is specific to our particular autistic sample. What remains to be explored is the extent to which the VPT performance in our autistic adults might be limited to a high functioning subset of autistic individuals (cf. Scheeren et al., 2013). Indeed, simply by attending university our autistic sample already represent a minority of individuals with an autism diagnosis (Helles et al., 2016; Newman et al., 2011). Future research should explore whether factors such as level of education or specific autistic diagnostic categories predict VPT abilities across the full range of the autism spectrum.

Finally, we have interpreted the effects of the actor’s looking and reaching upon VPT performance as indicative of participants using an EET strategy. This interpretation follows how such effects have been explained in the VPT literature (Furlanetto et al., 2013; Tversky & Hard, 2009) and how the absence of such effects has been interpreted in other studies with autistic participants (Conson et al., 2015). However, the link between sensitivity to another agent’s behaviour when making a VPT judgement, and the actual cognitive processes engaged by that judgement (e.g., MR or EET), is currently more based on theoretical interpretations than a substantial body of empirical evidence. Future research could more directly measure strategy use by, for example, correlating performance on MR and bodily transformation tasks with VPT performance in both autistic and non-autistic adults, as has been done with young children (Pearson et al., 2014; for similar arguments see also Pearson et al., 2016). Indeed, if sensitivity to social cues is to be interpreted as a measure of EET strategy use, then it would be valuable to know whether the magnitude of the social cue effect within participants (i.e., the extent to which a given participant’s perspective taking performance is affected by seeing an actor reach for an object) correlates with measures such as their bodily transformation abilities (cf. Pearson et al., 2016) and other suggested indices of EET strategy use (e.g., the extent to which one’s own body position affects VPT performance: Surtees et al., 2013a, b). Such data would allow for firmer conclusions regarding the exact processes underlying the effects reported here, and a better understanding of the implications of such conclusions with respect to the profile of VPT abilities in autistic adults.

To sum up: across two experiments, we investigated autistic and non-autistic adults visual perspective taking abilities—reporting the location of an object from their own perspective, or that of an actor. Both groups showed sensitivity to the behaviour of the actor, more accurately reporting his perspective when he was grasping and gazing toward the object compared to when he was not. In contrast to the recent findings of Conson et al. (2015) no group differences were observed, adding to the growing literature suggesting similar VPT level 2 abilities in autistic and non-autistic adults.

## Supplementary Information

Below is the link to the electronic supplementary material.

Supplementary file1 (DOCX 26 kb)
