# Supplementary material for: Autistic Adults Show Similar Performance and Sensitivity to Social Cues on a Visual Perspective Taking Task as Non-autistic Adults
Source: J Autism Dev Disord. 2022 Feb 19;53(5):1781–94. doi: 10.1007/s10803-022-05480-8 (PMC10123028; doi:10.1007/s10803-022-05480-8)
Supplement: Supplementary file 1 — Supplementary file1 (DOCX 26 kb) [file 10803_2022_5480_MOESM1_ESM.docx]

**Non-parametric analyses**

*Experiment 1*

*Error Data*

To investigate differences between the autistic and control groups, pair-wise Mann-Whitney U tests were performed on each of the four experimental conditions. In all cases no significant differences were found between groups (self: no-cue, Z = .46, p = .64; self: cue, Z = 1.30, p = .19; other: no-cue, Z = .85, p = .40; other: cue, Z = .55, p = .58).

To investigate differences according to the presence of the social-cue, pair-wise Wilcoxon signed-rank tests were performed comparing no-cue to cue conditions within each of the perspective conditions, separately for the autistic and control groups. In the autistic group no differences were observed between no-cue and cue conditions in either self (Z = .57, p = .57) or other (Z = .54, p = .59) perspective conditions. In the control group, no differences were observed between no-cue and cue conditions in either self (Z = 1.06, p = .29) or other (Z = .42, p = .68) perspective conditions.

To investigate differences according to the perspective taken, pair-wise Wilcoxon signed-rank tests were performed comparing self to other perspectives within each of the social-cue conditions, separately for the autistic and control groups. In the autistic group no differences were observed between self and other perspectives in either the no-cue (Z = .99, p = .32) or cue (Z = .31, p - .76) conditions. In the control group, no differences were observed between self and other perspectives in either the no-cue (Z = .22, p = .83) or cue (Z = .87, p = .38) conditions.

The above non-parametric analysis therefore confirms the outcomes of the parametric analysis reported in the main text: on error rates, there were no effects according to condition nor group.

*RT Data*

To investigate differences between the autistic and control groups, pair-wise Mann-Whitney U tests were performed on each of the four experimental conditions. Significant, or marginally significant, differences were observed between groups in all four conditions (self: no-cue, Z = 2.34, p = .019; self: cue, Z = 2.12, p = .034; other: no-cue, Z = 2.57, p = .009; other: cue, Z = 1.88, p = .062), with faster RTs in the control group across all four conditions.

To investigate differences according to the presence of a social-cue, pair-wise Wilcoxon signed-rank tests were performed comparing no-cue to cue conditions within each of the perspective conditions, separately for the autistic and control groups. In the autistic group no differences were observed between no-cue and cue conditions in either self (Z = 1.23, p = .22) or other (Z = 1.35, p = .18) perspective conditions. In the control group, no differences were observed between no-cue and cue conditions in either self (Z = 1.63, p = .10) or other (Z = 1.30, p = .19) perspective conditions.

To investigate differences according to the perspective taken, pair-wise Wilcoxon signed-rank tests were performed comparing self to other conditions within each of the social-cue conditions, separately for the autistic and control groups. In the autistic group, RTs were faster for self perspective judgements compared to other perspective judgements in both the no-cue (Z = 3.20, p = .001) and cue (Z = 3.48, p < .001) conditions. Similarly, in the control group RTs were faster for self perspective judgements compared to other perspective judgements in both the no-cue (Z = 3.53, p < .001) and cue (Z = 3.43, p < .001) conditions.

To investigate whether the magnitude of this perspective effect on RTs was different between the two groups, other – self perspective RT differences were calculated for each participant. Given the lack of effects of social-cue, these were calculated by first collapsing across no-cue and cue conditions, and then taking the difference between other and self perspectives. A Mann-Whitney U test found no difference between the autistic group and the control group in these other – self perspective RT differences (Z = .81, p = .42).

The above non-parametric analysis confirms the outcomes of the parametric analyses (ANOVAs) reported in the main text. On RT, overall the control group was faster than the autistic group. Both groups showed an effect of perspective on RTs, with self perspective judgements performed faster than other perspective judgements. The size of this effect did not significantly differ between the two groups. Finally, there was no effect of social-cue on RT.

*Experiment 2*

*Error Data*

To investigate differences between the autistic and control groups, pair-wise Mann-Whitney U tests were performed on both experimental conditions. No significant differences were found between groups in both the no-cue (Z = .99, *p = .32*) and the cue (Z = .39, p = .69) conditions.

To investigate differences according to the presence of the social-cue, pair-wise Wilcoxon signed-rank tests were performed comparing no-cue to cue conditions separately for the autistic and control groups. Marginally significant differences were observed between no-cue and cue conditions in both the autistic (Z = 1.90, p = .058) and control group (Z = 1.84, p = .065), with fewer errors in the cue condition compared to the no-cue condition. To investigate whether the magnitude of this social-cue effect on ER was different between the two groups, no-cue – cue ER differences were calculated for each participant. A Mann-Whitney U test found no difference between the autistic group and the control group in these no-cue – cue ER differences (Z = .97, p = .33). Finally, it should be noted that while within each group the effect of cue was marginally significant, when taking the full sample as a whole there was a significant overall effect of cue (Z = 2.50, p = .013).

The above non-parametric analysis therefore confirms the outcomes of the parametric analyses (ANOVAs) reported in the main text. Overall there was an effect of cue across both groups (i.e., a main effect of cue), with evidence for this effect in each group considered separately and no significant difference in the size of this effect between groups.

*RT Data*

To investigate differences between the autistic and control groups, pair-wise Mann-Whitney U tests were performed on both experimental conditions. No significant differences were found between groups in both the no-cue (Z = .31, p = .76) and the cue (Z = .15, p = .90) conditions.

To investigate differences according to the presence of the social-cue, pair-wise Wilcoxon signed-rank tests were performed comparing no-cue to cue conditions, separately for the autistic and control groups. No differences between no-cue and cue conditions were observed in either the autistic group (Z = 1.62, p = .11) nor the control group (Z = .46, p = .65).

The above non-parametric analysis therefore confirms the outcomes of the parametric analyses (ANOVAs) reported in the main text: on RT there were no effects according to condition nor group.
